# Sir Victor Horsley

**Published:** 1916-07-29

**Authors:** 


### SIR VICTOR HORSLEY.

### A Man of Genius and Personality.

The death of Sir Victor A. H. Horsley, F.R.S., F.R.C.S., in Mesopotamia from sunstroke, which we announced in last week's issue, removes one whose personality was perhaps the most arresting and unique in London surgical circles: not a perfect man nor a pleasant one, but for all that a man of real genius and of great achievements. The outline of his professional and institutional career was given in these pages last week; of the man himself there is something more to be said. It is no exaggeration that Horsley's association with the National Hospital for the Paralysed and Epileptic, Queen Square, had much to do with that hospital's reputation as the premier institution of its class in the world. By his bold and daring researches in the 'eighties and 'nineties, Horsley created cranial surgery, and laid its foundation so well and truly that all other workers since then have been content to build upon them. Nor was Horsley just an experimenter, a discoverer of principles, an explorer in a new region of surgery; he remained for thirty years and more not only the pioneer of brain and spinal-cord surgery, but also by far its most brilliant craftsman. There arrived in time others whose opinion was sounder as to whether or not an operation should be done; there was none whose technique in a dangerous or difficult case was so masterly. Probably it was the consciousness of this technical superiority that led him to operate on inoperable cases refused by other surgeons. It was certainly not through wilful carelessness of the welfare of his patients that he operated too much, as undoubtedly he did.

### The Sport of Destiny.

But surgery by no means filled up Horsley's busy life, though his achievements in that sphere were far more important than anything he attained elsewhere. His masterful, restless temperament, his intense energy and earnestness, his impatience of compromise, and his love of strife continued to form a personality as strange and as strongly marked as any of modern times. Indeed, the relentlessness and irony of his fate were more like those of a Greek tragedy than of a modern Englishman; and the climax of his death in Mesopotamia suggests the art of Æschylus or Sophocles more than sober truth. Horsley, the greatest cerebral surgeon in the world, struck down by a cerebral disease; struck down, moreover, while serving his country in a war which he and the advanced wing (to which he belonged) of the Party in power had by their pacifist and anti-preparedness policy done so much to bring about.

Nor was it only in death that he seemed to be the sport of Destiny. When he sought Parliamentary honours, chance (in the shape of the Party organisers, presumably) allotted him to a constituency in the Leicester area, full of advanced Radicals many of whom happened also to be anti-vivisectionists and anti-vaccinators. The arch-vivisector of modern times, whose researches were based first and last on vivisection, tied by political ambition to men who hated vivisection even more than they hated the House of Lords! The most tactful of reconcilers, the most adroit of Party coachmen, would have found the position intolerable: Horsley detested compromise as much as his supporters loathed vivisection, and after a stormy year or two his candidature was withdrawn. So again with woman's suffrage, of which he was a vehement champion. When the "hunger strikes" were the talk of the town, Horsley rushed into the fray with pronouncements upon forcible feeding which showed that his sympathy with the suffrage cause had quite carried away his medical judgment. Not only did his own words make him ridiculous to his medical colleagues, but he had to be reminded that his treatment of his own nurses was violent and overbearing to absolute brutality, as in fact it frequently was.

### Social Reform.

So again with medical politics: struck with the inefficient organisation of the profession, he set to work to improve the machinery of the British Medical Association, and did so with much success. For this he certainly deserved the thanks of all medical men, and for a time he had them; but he could not resist the temptation to use the weapon he had forged for the furtherance of his own views, and those of Mr. Lloyd George, upon social reform, and the upshot was the furious resentment of the bulk of the profession. Amongst his other striking qualities, courage was pre-eminent in Horsley's character, and the scene some six or seven years ago when he faced about fifteen hundred incensed doctors in Queen's Hall will not soon be forgotten by anyone who was present. He confronted the meeting as coolly as if he were trepanning a patient or vivisecting an ape; and if he did not convince it that he had been justified in his course of action, at least he secured a hearing when few men in his position could have done so.

### An Untiring Worker.

Institutionally, too, Horsley was a difficult problem. He would not recognise the right of lay boards to control expenditure which affected surgical treatment, and troubles naturally resulted. But when all is said and done, he was the greatest figure in British surgery between Lister and the end of the nineteenth century. With all his defects, he was a man of real genius. Since he was also an untiring worker, his work has stood the test of time, as time is reckoned in surgery, and his name must unquestionably be inscribed on the select roll of those whose labours have contributed to the permanent welfare of their fellow-men. He was in many ways very un-English; but there is no denying that he was—what always appeals to English ideas—emphatically a great man.

